# Antioxidant Potential, Phytochemicals Composition, and Metal Contents of* Datura alba*

**DOI:** 10.1155/2019/2403718

**Published:** 2019-06-17

**Authors:** Waliullah Khan, Sidra Subhan, Dilawar Farhan Shams, Sahib Gul Afridi, Riaz Ullah, Abdelaaty A. Shahat, Ali S. Alqahtani

**Affiliations:** ^1^Department of Chemistry, Abdul Wali Khan University, Mardan, Pakistan; ^2^Department of Environmental Sciences, Abdul Wali Khan University, Mardan, Pakistan; ^3^Department of Biochemistry, Abdul Wali Khan University, Mardan, Pakistan; ^4^Medicinal, Aromatic and Poisonous Plants Research Center (MAPRC), College of Pharmacy, King Saud University, P.O. Box 2457, Riyadh 11451, Saudi Arabia; ^5^Phytochemistry Department, National Research Centre, P.O. Box 1262233, El Bohouth St., Dokki, Giza, Egypt; ^6^Department of Pharmacognosy, College of Pharmacy, King Saud University, P.O. Box 2457, Riyadh 11451, Saudi Arabia

## Abstract

This study investigated the phytochemical characteristics and antioxidant activity in leaves, roots, stem, flower, and seed parts of* Datura alba (D. alba). *The study also assessed the heavy metal (Cr, Mn, Zn, and Cu) accumulation in each part of the plant. Among the phytochemicals, alkaloids were found only in leaves while tannins, flavonoids, and phenols were present in all parts of the plant. For antioxidant activity, free radical scavenging assay for 2,2-diphenyl-1-picrylhydrazyl (DPPH) was performed using ascorbic acid as the standard. Higher activity was shown by stem extract in methanol and leaf extract in n-hexane, ethyl acetate, and chloroform. Furthermore, all the target heavy metals were detected in all plant sections with the highest concentration of Zn in leaves and Cu in stem, root, flower, and seed. Due to stronger antioxidant potential and phytochemical composition,* D. alba* could prove as valuable prospect in pharmaceutical formulations by taking part in the antioxidant defense system against generation of free radicals.

## 1. Introduction

A great number of modern medicines have been derived from plants that are considered as important sources of medicinal agents to treat different diseases [1]. For drug development, bioactive compounds like flavonoids, tannins, phenols, and alkaloids in medicinal plants play a vital role [2].* Datura* is a shrub-like perennial herb containing 15 species, of which* D. alba* is considered as the most important drug plant for its chemical and ornamental properties. It belongs to the family Solanaceae and is commonly known as Devil's trumpet or Angel's trumpet with an average height of 1.5m [3]. This plant is widely used in medicines to treat diseases like cough, asthma, diarrhea, epilepsy, insanity, rheumatic pains, catarrh, hysteria, and skin ulcers [4].* D. alba *is also a heavy-metal-tolerant plant that can grow easily in metal polluted sites. Its hyperaccumulative nature has been exploited for the recovery of toxic heavy metals from contaminated ground water [5].

For normal cellular activities, a balance between the antioxidant defense mechanism and the produced reactive oxygen species (ROS) is very important. An increase in ROS disturbs this equilibrium and results in oxidative stress [6]. Although ROS, which are produced from partial reduction of oxygen during high metabolism, are important for life, repeated stress conditions lead to increase in energy utilization and hence production of more ROS, which may harm cells, tissues, and organs. Several plant extracts possess significant antioxidant activities to treat many diseases and disorders such as liver toxicity, diabetes hepatotoxicity, and other complications [7].

Phytochemicals derived from plants are nonnutritional natural compounds that are important for numerous body functions in humans. Many of these compounds found in food products are known to have antioxidant potential due to the occurrence of OH group [8]. The antioxidants prohibit the oxidative damage to various macromolecules like nucleic acids, proteins, and lipids and scavenge free radicals generated from biochemical reactions [9]. A reaction of these free radicals with macromolecules has been reported to stimulate apoptosis that may cause various physiological, cardiovascular, and neurological disorders [10]. Various types of phytochemicals such as phenolic acids, ascorbic acid, tocopherols, and bioflavonoids having antioxidant characteristics have been used to treat many diseases [11].

Keeping in view the importance of* D. alba *in traditional medicines and the existence of bioactive compounds in various parts of this plant, this study was intended to investigate its phytochemical and pharmacological properties. Earlier studies have mainly used hydroalcoholic and ethanol extracts for phytochemical and antioxidant analysis of* D. alba *[12, 13]. This study investigated the crude phytochemicals (qualitative and quantitative), antioxidant activities, and heavy metals in* D. alba *using different types of solvents (polar and nonpolar). Also, the study was aimed at boosting the scientific database on the subject medicinal herb.

## 2. Materials and Methods

### 2.1. Preparation of Crude Extracts

The plant samples were collected from District Charsadda, Khyber Pakhtunkhwa, Pakistan. The plant specimen was identified at the Department of Botany, Abdul Wali Khan University Mardan, Pakistan, following the flora of Pakistan. The plant was assigned voucher number AWKUM.Bot.168.10.9:44-45 and was placed at the Department of Botany's herbarium. The plant extracts were made following the standard procedures [14–16].

### 2.2. Determination of Phytochemicals

Powdered samples (5g) were added in 100mL of distilled water and kept for two days at room temperature for complete extraction. After two days, the extracts were filtered through Whatman filter paper (Grade 41). Qualitative and quantitative phytochemicals analyses for each sample were performed using the methods available in literature [17–20].

#### 2.2.1. Alkaloids Test

For alkaloid test, HCl (1%) was added to 3mL of aqueous extract and then heated on water bath at 100°C. The extract was then cooled and a few drops of Mayer reagent were added. The sample was kept for 5min and observed for turbidity, which indicated the presence of alkaloids [21].

#### 2.2.2. Flavonoids Test

For flavonoids test, 10% lead acetate solution (1mL) was added to 1mL of each extract. The solution was allowed for few minutes until yellow precipitate was formed [22].

#### 2.2.3. Tannins Test

For tannins test, 1 to 2 drops of ferric chloride (FeCl_3_) solution and 1mL of distilled water were added to 1mL of the extract. The extract solution was thoroughly mixed and left for few minutes until formation of a blue or green black coloration [23, 24].

#### 2.2.4. Phenols Test

For phenols test, 2mL of ethanol and few drops of FeCl_3_ solution were mixed in the extract solution and allowed for few minutes. The change in color to black indicated the presence of phenols [23, 24].

### 2.3. Quantitative Analysis of Phytochemicals

#### 2.3.1. Alkaloids Content

For alkaloids content, 100mL of acetic acid (10%) in ethanol was first mixed with 5g of dry plant sample and then covered and allowed for 4hrs to complete the extraction process. This was followed by filtration and then concentrating the filtrate using a water bath to 1/4th of the initial volume. Concentrated ammonium hydroxide (NH_4_OH) was then added until the formation of precipitate, which was then filtered, washed several times with diluted NH_4_OH, and dried to obtain the alkaloid residue.

#### 2.3.2. Flavonoids

Flavonoids in various parts of* D. alba* were analyzed using the method described by Natic et al. [25].

#### 2.3.3. Tannins

The total content of tannins was determined following the method by Medini et al. [26] with little modifications. To 50mL of distilled water, 500mg of plant sample was added and stirred for 1hr. Afterwards, the solution was filtered and made up to 50mL. Then 2mL of 0.1M FeCl_3_ solution was added to 5mL of the filtered sample into a test tube and the absorbance was measured within 10min at 395nm using UV-5100B UV/VIS spectrophotometer.

#### 2.3.4. Phenols

Total phenolic content was determined quantitatively following Oyewole and Akingbala [27]. The powdered plant sample (3g) was boiled with 30mL of ether in a condenser for 15min at 34°C and then filtered to obtain the ether extract. To 50mL volumetric flask, 5mL of extracted sample, 5mL of concentrated isoamyl alcohol, and 2mL of NH_4_OH solution were added and the final volume was made up to 50mL with distilled water. The solution was left for 30min until the color change. Finally, the absorbance was measured at 505nm using UV/VIS spectrophotometer.

### 2.4. Total Antioxidant Capacity Assessment

The DPPH method was used to assess the total antioxidant capacity by measuring the DPPH assay according to Rivero-Perez [28]. DPPH is a commercially available nitrogen free radical with dark purple color showing maximum absorbance at 517nm. When antioxidant molecules (methanol extracts) were incubated at room temperature with DPPH, the DPPH radical reduced with the transfer of hydrogen from antioxidant molecule, resulting in color change from dark purple to yellow and the absorbance of DPPH decreased. The DPPH scavenging activity was determined for various extracts of n-hexane, ethyl acetate, methanol, and chloroform. Ascorbic acid and methanol were used as standard and blank solvent, respectively. Stock solutions (25mg/mL) were prepared in methanol from each extract. Working solutions (10, 20, 30, 40, and 50*μ*g/mL) were prepared from these stock solutions. DPPH solution (0.96mM) was also prepared in the same solvent. Thereafter, 1mL of DPPH solution was mixed with each working solution followed by incubation for 30min at room temperature. Control was prepared by adding 1mL DPPH to 4mL methanol. The absorbance of samples and the control was measured at 517 nm using UV/VIS spectrophotometer. The DPPH scavenging activities were measured as percent inhibition using the following equation:

(1) Inhibition  %=Absorbance  Control−Absorbance  test  sampleAbsorbance  Control×100

The IC_50_ value of sample was obtained by linear regression analysis of concentrations and inhibition (%).

### 2.5. Determination of Heavy Metals

#### 2.5.1. Digestion of Plant Parts

Analytical grade concentrated per chloric acid (HClO_4_) and nitric acid (HNO_3_) were used for the digestion. Samples (0.2g) of dry grounded plant parts (root, stem, leaves, seeds, and flowers) were weighed into 100mL beakers. Predigestion of the samples was performed with HNO_3_ (5mL), followed by cooling and digestion again to HC1O_4_ fumes [29]. After digestion, distilled water (50mL) was added and the mixture was nearly boiled to achieve complete dissolution. After cooling, the samples were filtered using Whatman filter paper (Grade 41).

#### 2.5.2. Heavy Metal Analysis

Heavy metals analyses for zinc (Zn), copper (Cu), manganese (Mn), and chromium (Cr) were performed in triplicates for all parts of medicinal plants using atomic absorption spectrophotometer (Analyst 700, Perkin Elmer).

## 3. Results

### 3.1. Phytochemical Analysis

**Figure 1:**
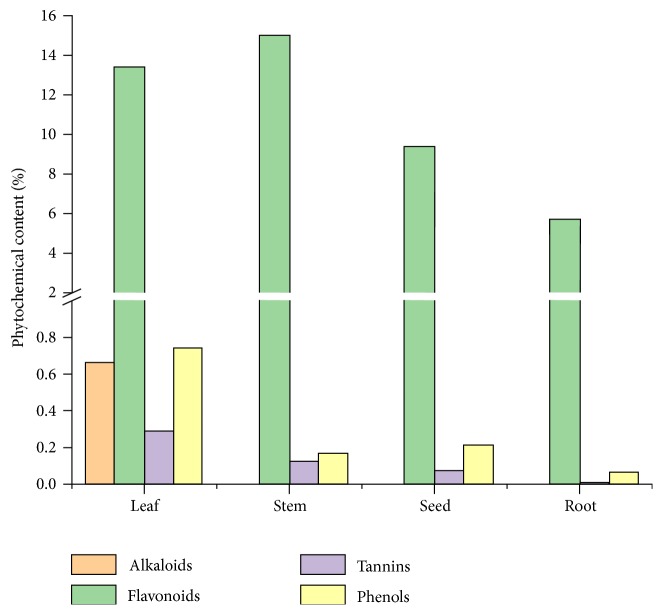
Phytochemicals content in different parts of* D. alba.*

The phytochemical analysis of* D. alba* showed the presence of alkaloids, flavonoids, tannins, and phenols. Qualitative analysis of phytochemicals in various plant extracts showed the presence of flavonoids, tannins, and phenols in all the four parts (leaf, stem, seed, and root), while alkaloids were found only in leaf (Table 1). This was in line with the literature as the maximum amounts of alkaloids and bioactive compounds are present in leaves [30]. Quantitative analysis of phytochemicals is shown in Figure 1.

### 3.2. Determination of Antioxidant Activity

**Figure 2:**
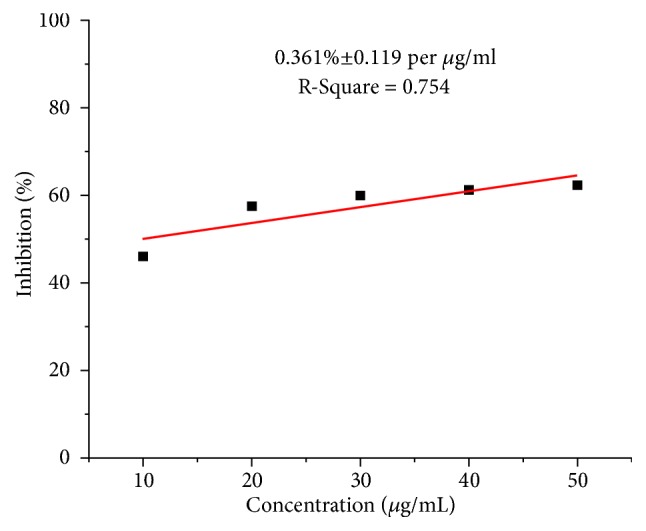
Antioxidant activity of ascorbic acid.

The DPPH scavenging activity of the various parts of* D. alba *extracts in different solvents at concentrations varying from 10 to 50*μ*g/mL was determined with Vit. C (ascorbic acid) as standard.  Figure 2 indicates the inhibition (%) as a function of Vit. C concentration. The results revealed that, with the increasing Vit. C concentration, the percentage inhibition (%) increased at a rate of 0.361%±0.119 per *μ*g/mL of Vit. C, while the maximum activity was attained at 50*μ*g/mL.

#### 3.2.1. Antioxidant Activity of Methanol Extracts

**Figure 3:**
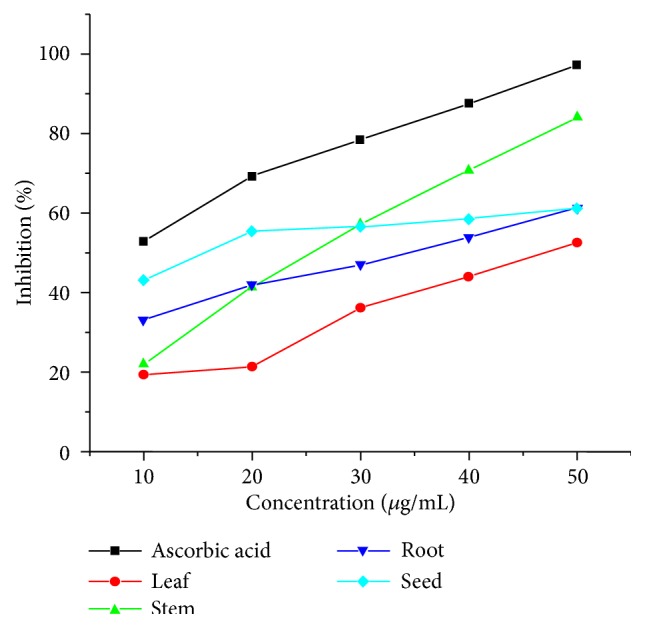
Antioxidant activity of methanol extracts.

Free radical scavenging assay was used to determine the antioxidant activity of methanol extracts of root, stem, leaf, and seed parts of* D. alba *(Figure 3). A concentration range of 10-50*μ*g/mL was used. According to results, the standard ascorbic acid showed the maximum antioxidant activity at all concentrations (10-50*μ*g/mL) in comparison to the stem, root, leaf, and seed extracts. The percent inhibition of stem extract was 57.19%, 70.78%, and 84.05% at a concentration of 30, 40, and 50*μ*g/mL, respectively. Seed extracts showed higher activity at lower concentration; root extracts exhibited moderate activity, while leaf extracts showed low activity.

#### 3.2.2. Antioxidant Activity of n-Hexane Extracts

**Figure 4:**
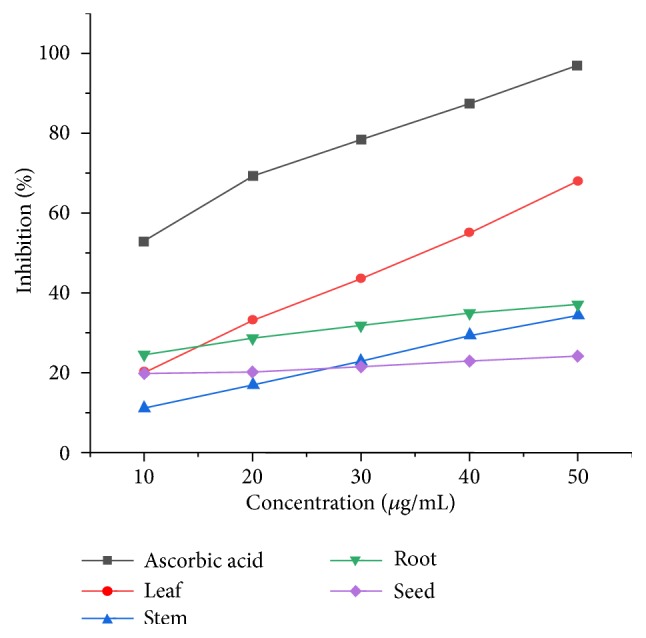
Antioxidant activity of n-hexane extracts.

As shown in Figure 4, the n-hexane extracts of all parts of* D. alba* showed antioxidant activity that decreased in the following order: leaf (68%) > root (37%) > stem (34%) > seed (24%) by the DPPH free radical scavenging method.

#### 3.2.3. Antioxidant Activity of Ethyl Acetate Extracts

**Figure 5:**
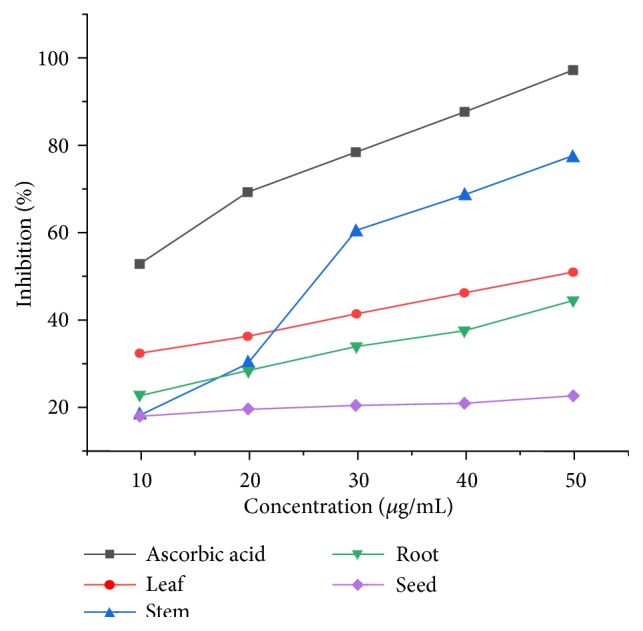
Antioxidant activity of ethyl acetate extracts.

Antioxidant activities for various parts of* D. alba *in ethyl acetate are shown in Figure 5. From the IC_50 _values, it is evident that extract of seeds had the lowest inhibition activity, whereas that of leaf possessed the highest inhibition activity. The antioxidant activity for the ethyl acetate extracts decreased in the order of leaf > root > stem > seed.

#### 3.2.4. Antioxidant Activity of Chloroform Extracts

**Figure 6:**
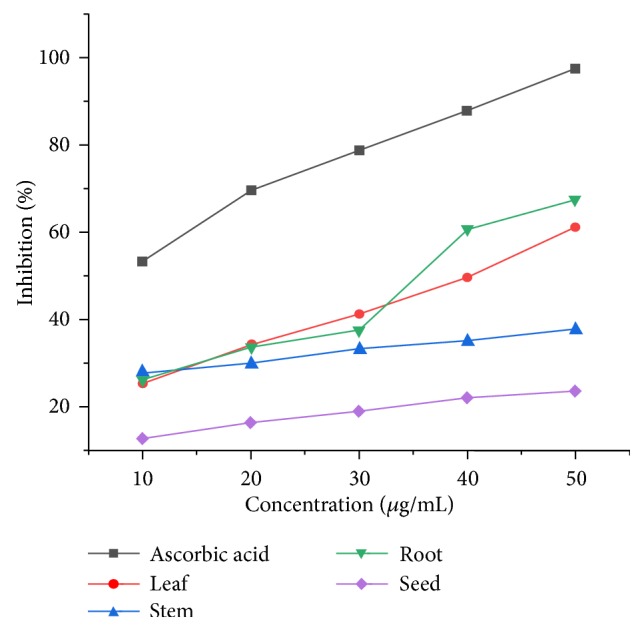
Antioxidant activities of chloroform extracts.

In chloroform extracts, root extract had high antioxidant activity at 40*μ*g/mL and 50*μ*g/mL and leaf at 30*μ*g/mL (Figure 6). Similarly, the stem also showed high activity at low concentration of 10*μ*g/mL but moderate activity at all other tested concentrations. As a whole, the root section showed high antioxidant activity at higher concentrations and the leaf at lower concentrations.

#### 3.2.5. Antiradical Activity on DPPH (IC_*50*_)

**Figure 7:**
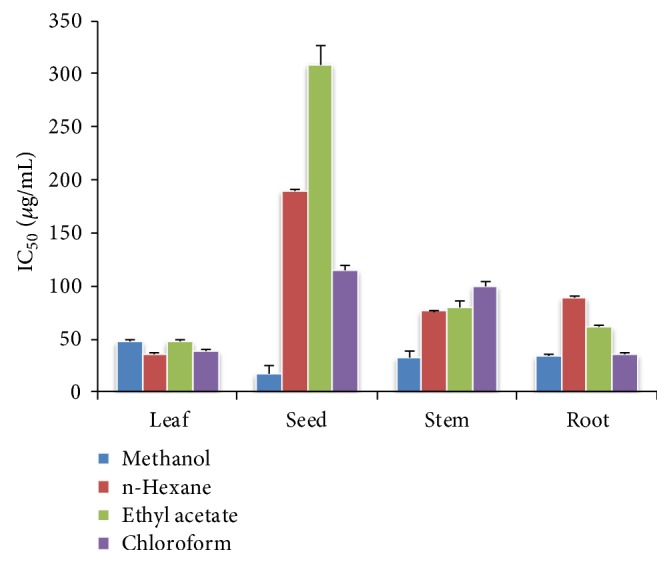
The IC_50_ values for leaf, seed, stem, and root extracts in methanol, n-hexane, ethyl acetate, and chloroform.

IC_50_ is the concentration at which the inhibition is 50%. It has an inverse relationship to antioxidant activity. The decreasing order of antiradical activity was Vit. C > methanol > chloroform > n-hexane>ethyl acetate.  Figure 7 shows the IC_50_ values for various extracts in various parts of the plant in the following order: leaf < root < seed < stem.

### 3.3. Determination of Heavy Metals

Heavy metal analysis in various parts of the plant revealed that the leaf part contained high concentration of Zn, while Cu was found in comparatively higher concentration in all parts of the plant (Table 2). Mn was present in the lowest amounts among the target heavy metals. High concentration of Cu was found in flower of* D. alba *(4.23±0.25ppm) followed by seed (3.51±2.45ppm), leaf (2.83±0.95ppm), root (2.83±0.15ppm), and stem (2.48±0.15ppm), respectively (Table 2). Maximum concentration of Mn was found in the leaf (0.74±0.01ppm) followed by root (0.25±0.01ppm), flower (0.19±0.01ppm), stem (0.18±0.01ppm), and seed (0.10±0.00ppm). These results were within the limits of WHO. In case of Cr, root has a concentration of 2.06±0.05ppm, seed 1.42±0.06ppm, stem 1.31±0.37ppm, flower 1.30±0.30 ppm, and leaf 0.98±0.07ppm. Highest levels of Cr were found in root and the lowest in leaves.

## 4. Discussion

### 4.1. Phytochemical Analysis

The overall concentration of flavonoids in all parts of the plant was more than phenols and tannins and this corresponded with the findings by Genskowsky et al. [31]. Alkaloids are a special group of secondary nitrogenous compounds. Plants containing alkaloids were used during the middle ages for various human and animal diseases [32]. Most of the plants contain flavonoids that constitute a large number of polyphenolic compounds. They are a key component of human dietary intake that could be used in curing cardiovascular dysfunctions, cancer, and inflammatory diseases [33].

Tannins are polyphenolic compounds with higher molecular weight which are present in many plants and are known to provide protection against microorganisms. In animals, tannins may cause indigestion of proteins and ultimately retardation of animal growth due to formation of hydrogen bonds with carboxylic groups [34]. Meanwhile, phenolic compounds consist of aromatic ring (with the phenyl hydroxyl or its substituted radicals), which are plant secondary metabolites having little contribution towards the physiological or ecological functions of the plant [35].

### 4.2. Determination of Antioxidant Activity

Plants are the main source of natural antioxidants as many of the phytochemicals have antioxidant properties due to hydroxyl groups in their structural formulae. Their main activity is to protect the defense system against oxidative stress by free radicals [36]. Various antioxidant compounds produced by plants stabilize ROS [37]. ROS are of two types, free radicals such as hydroxyl and superoxide anion radicals (O_2_^−^) and nonfree radicals, mostly activated oxygen like singlet oxygen (^1^O_2_) and H_2_O_2_. DPPH that has free radical scavenging activity was used in this assay. It is reduced by the transfer of hydrogen atom from antioxidant molecule resulting in the color change at specific wavelength.

The maximum absorbance for methanol extracts was obtained at 50*μ*g/mL for leaves, stem, root, and seed extracts, respectively. According to these results, stem extract exhibited the maximum antioxidant activity due to the presence of higher quantity of flavonoids (15%) as shown in Figure 1 [38]. Due to high flavonoid and phenolic content, the n-hexane extracts of leaves have relatively better reducing power and DPPH radical scavenging activity. It was also rich in flavonoids (13.4%) and phenolic content (0.742%), which is responsible for its comparatively higher antioxidant activity. The same for the ethyl acetate extracts decreased in the order of leaf > root > stem > seed. These results for chloroform extracts revealed the medicinal importance of the plant with all parts containing good amounts of flavonoids that are a source of antioxidant activity. It is also evident from the IC_50_ values that all parts of* D. alba *exhibited antioxidant activity.

### 4.3. Antiradical Activity on DPPH (IC_*50*_)

Plant extracts that have higher concentration of flavonoids have better capability to donate hydrogen atoms to the scavenging free radicals. This decreasing order of antioxidant activity showed that a lesser quantity of the extract having higher radical activity is needed to inhibit 50% of DPPH free radical, whereas a larger quantity is required for the lesser active extract. It is clear from Figure 6 that all the plant extracts were active species for antioxidant activity due to the existence of flavonoids, which can neutralize the free radicals to give stable compounds.

### 4.4. Determination of Heavy Metals

According to FAO/WHO [39], the permissible limit for Zn, Cu, Cr, and Mn is 27.4, 3.00, 0.02, and 2ppm, respectively. From the results, Zn concentration was within the standard limits. Zn is an essential trace nutrient for plant growth due to its role in various cell functions. It is also important for brain development, wound-healing, bone formation, normal growth, and behavioral response with a dietary limit in humans of 100ppm [40]. Its deficiency causes diabetes and loss of smell and touch. Cu is important for normal plant growth but its extreme levels (>100ppm) can cause phytotoxicity [41]. The concentration of Cu in seed and flower parts exceeded the prescribed WHO limits but its concentration was lower in leaf, stem, and root. Meanwhile, with respect to body weight, the acceptable lower limit of Cu is 20*μ*g/mg body weight per day [42]. The deficiency of Mn can cause chlorosis as it is also an essential nutrient for plants [43]. In adult humans, the suitable daily dietary intake of Mn is 11mg/day [44]. Mn deficiency causes disorder of bone development in children, which may result in rheumatic arthritis and immunodeficiency disorder in adults [45, 46]. In case of Cr, the values were higher than the permissible limits as suggested by FAO/WHO. The Cr intake causes toxic effects in humans like irritations of nose, skin rash, lungs cancer, and damage to kidney and liver, whereas its deficiency causes trouble in lipids, protein, and glucose metabolism [47]. For adults, the recommended daily intake of Cr is 50-200*μ*g/day [48].

## 5. Conclusion

Phytochemicals (tannins, phenols, alkaloids, and flavonoids) were found in all parts of the plant; however, the highest amounts were present in the leaf part of* D. alba*. The presence of these phytochemicals makes the plant antiradically active with the various parts (extracts) of* D. alba* showing antioxidant activity. Methanol showed higher antioxidant activity especially in the leaf extract due to the presence of high concentrations of polyphenolic constituent including flavonoids and tannins. Likewise, the plant contained heavy metals within the WHO/FAO permissible limits except for chromium. From the presence of phytochemicals, antioxidant activity of the plant, and availability of micro and macro elemental nutrients, it could be deduced that* D. alba *could be a prospective addition in pharmaceutical products to improve human health by participating in the antioxidant defense system against production of free radical.

## Figures and Tables

**Table 1:** Qualitative analysis of phytochemicals.

Sample	Alkaloids	Flavonoids	Tannins	Phenols
Leaf	**++**	**+ +**	**+ +**	**+ +**
Stem	- -	**+ +**	**+ +**	**+ +**
Seed	- -	**+ +**	**+ +**	**+ +**
Root	- -	**+ +**	**+ +**	**+ +**

+ +: present; - -: absent.

**Table 2:** Heavy metal concentration (ppm) in various parts of *D. alba *(Mean ± SD).

Sample	Cu	Cr	Zn	Mn
Leaf	2.83±0.95	0.98±0.05	5.98±0.03	0.74±0.01
Stem	2.48±0.15	1.31±0.37	1.51±0.02	0.18±0.01
Flower	4.23±0.25	1.30±0.30	1.93±0.04	0.19±0.01
Root	2.83±0.15	2.06±0.05	0.85±0.01	0.25±0.01
Seed	3.51±2.45	1.42±0.06	1.89±0.35	0.10±0.00

## Data Availability

The data used to support the findings of this study are included within the article.
